# Genomewide transcriptomic profiling identifies a gene signature for predicting recurrence in early‐stage hepatocellular carcinoma

**DOI:** 10.1002/ctm2.405

**Published:** 2021-06-06

**Authors:** Tatsuhiko Kakisaka, Moto Fukai, Jasjit K. Banwait, Toshiya Kamiyama, Tatsuya Orimo, Tomoko Mitsuhashi, Kensuke Yamamura, Takeo Toshima, Hideo Baba, Akinobu Taketomi, Ajay Goel

**Affiliations:** ^1^ Center for Gastrointestinal Research Center from Translational Genomics and Oncology Baylor Scott and White Research Institute and Charles A. Sammons Cancer Center Baylor University Medical Center Dallas Texas USA; ^2^ Department of Molecular Diagnostics and Experimental Therapeutics Beckman Research Institute of City of Hope Comprehensive Cancer Center Duarte California USA; ^3^ Department of Gastroenterological Surgery I Graduate School of Medicine Hokkaido University Hokkaido Japan; ^4^ Department of Surgical Pathology Hokkaido University Hospital Hokkaido Japan; ^5^ Department of Gastroenterological Surgery Graduate School of Medical Science Kumamoto University Kumamoto Japan; ^6^ Department of Surgery and Science Graduate School of Medical Sciences Kyushu University Fukuoka Japan

**Keywords:** biomarker, gene panel, hepatocellular carcinoma, recurrence

**Abbreviations**
AJCC: American Joint Committee on Cancer
AUC: area under the curve
BCLC: Barcelona Clinic Liver Cancer
C1QTNF8: C1q‐tumor necrosis factor‐related protein 8
CI: confidence interval
CoxPH: Cox proportional hazards
EPR: early phase recurrence
FFPE: formalin‐fixed paraffin embedded
GSE: gene expression omnibus series
HCC: hepatocellular carcinoma
HERC5: HECT and RLD domain containing E3 ubiquitin protein ligase 5
HR: Hazard ratio
LASSO: least absolute shrinkage and selector operation
qRT‐PCR: quantitative real‐time reverse transcription polymerase chain reaction
ROC: receiver operating characteristic
SNX24: sorting nexin 24
TCGA: The Cancer Genome Atlas

Dear Editor,

A majority of patients with hepatocellular carcinoma (HCC) suffer from tumor recurrence even after curative treatments. Such events can be categorized as either the early phase (within 2 years of treatment) or the late phase recurrence (after 2 years).^1^ The prognosis for patients with early phase recurrence (EPR) is generally much worse than the other group.^2^, ^3^, ^4^ As per the AJCC guidelines (7th edition) and the BCLC recommendations, an early‐stage HCC with an AJCC‐TNM stage‐I and BCLC stage‐0/A is defined by the following features: a solitary tumor, 5 cm or smaller in size, and a cancer without pathological vascular invasion. Accordingly, perhaps ∼25% of the early‐stage HCCs is likely at risk for developing EPR following curative treatment.^5^ Unfortunately, currently this is lack of availability of clinically useful biomarkers for predicting EPR in patients with early‐stage HCCs.^4^, ^6^ In the present study, we for the first time undertook a systematic and comprehensive biomarker discovery and validation approach to unravel a novel gene expression signature for detecting an EPR in early‐stage HCC patients.

Our study design included multiple biomarker discovery and validation phases (Figure S1). We first analyzed TCGA RNA‐Seq cohort comprising 59 early‐stage HCC patients with (*n* = 21) and without EPR (*n* = 38). Among the 20,502 transcripts, 342 genes were differentially expressed. Following exclusion of highly correlated genes, the association of each gene with recurrence‐free survival was assessed using Cox's proportional hazards (CoxPH) regression model and LASSO regression analysis, which resulted in a panel of eight‐candidate genes (Figure 1A), which were not correlated with each other (Figure 1B). We next plotted the receiver operating characteristic (ROC) curves in TCGA cohort. This eight‐gene panel accurately predicted EPR (the area under the curves [AUC] = 0.88; Figure 1C); which was subsequently validated in the GSE76427 cohort comprising 26 early‐stage HCC patients with (*n* = 15) and without EPR (*n* = 11; AUC = 0.81; Figure 1D). Patients who were categorized as high‐risk exhibited higher cumulative recurrence rates compared to the low‐risk group in both TCGA and GSE76437 cohorts (*p *< 0.0001 and *p *= 0.0030, respectively; Figures 1E and 1F).

**FIGURE 1:**
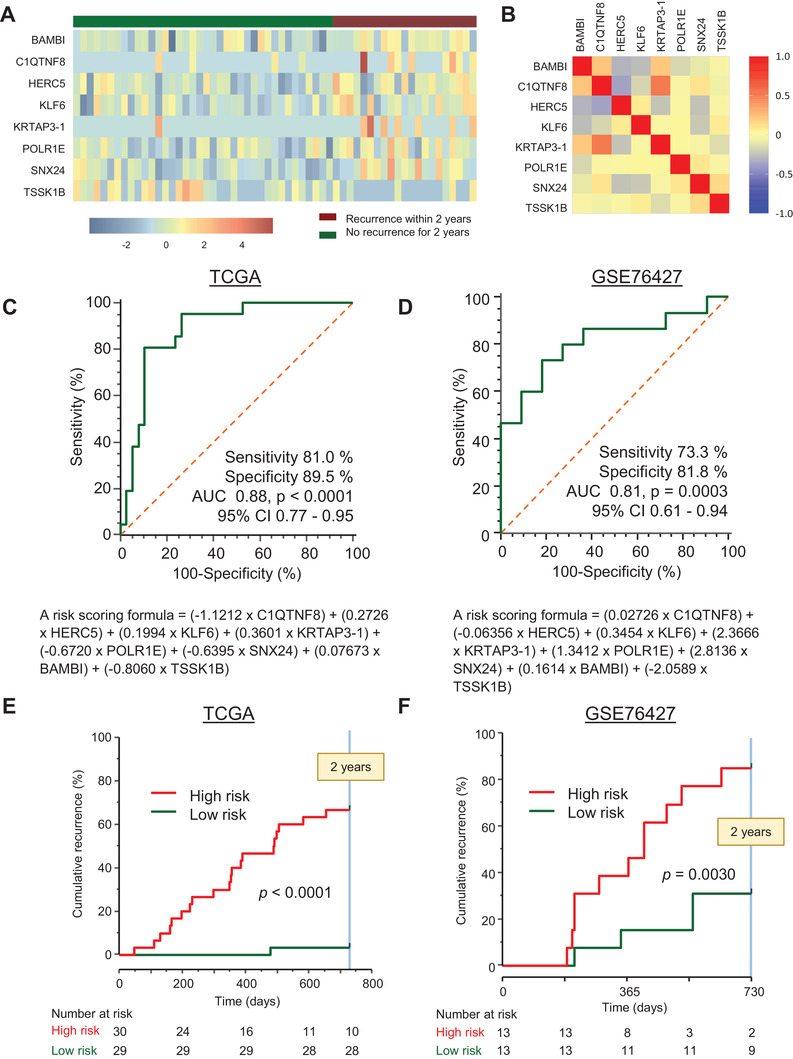
Predictive value of eight‐gene panel for identifying early phase recurrence in discovery and in‐silico validation cohorts. (A) A heatmap illustrating the expression levels of the eight candidate genes expressed differentially between patients with or without early phase recurrence in discovery (TCGA) dataset. (B) A correlation matrix of the selected eight genes in TCGA dataset. (C and D) Receiver operating characteristic (ROC) curves of discovery dataset (TCGA) and in‐silico validation dataset (GSE76427) for predicting early phase recurrence using eight‐gene panel, respectively. ROC curves are created by risk score based on a partial likelihood in Cox proportional hazard model for both TCGA and GSE76427 datasets individually. We used Youden's index for calculating sensitivity and specificity during ROC curve analysis. (E and F) Cumulative recurrence rate curves for detecting 2‐year recurrence in TCGA cohort and GSE76427 cohort using eight‐gene panel, respectively. Patients in each cohort are stratified into high‐ and low‐risk using median expression values of individual eight‐gene panel score as cutoff thresholds. Red and green lines indicate high‐risk and low‐risk patients, respectively

Next, we interrogated the performance of the eight‐gene panel in two independent clinical cohorts of 130 HCC patients (Table 1). In cohort‐1 (*n* = 54), 13 cases (24.1%) experienced EPR, while EPR occurred in 27 of 76 cases (35.5%) in the cohort‐2. We excluded the TSSK1B gene from further analysis due to low expression in FFPE tissues. We evaluated the expression of the remaining seven genes in the clinical cohorts using qRT‐PCR (Table S1). These experiments revealed that three genes (SNX24, C1QTNF8, and HERC5) were commonly expressed in both clinical cohorts (Figure S2) and hence were selected for further analysis. We thereafter confirmed the predictive accuracy of the three‐gene panel in TCGA and GSE76427 cohorts (Figure S3).

**TABLE 1:** Key clinical pathological features in the clinical training and testing cohorts of solitary HCC patients

	Cohort‐1 (*n* = 54)			Cohort‐2 (*n* = 76)		
	Recurrence within Two years (*n* = 13: 24.1%)	No recurrence for Two years (*n* = 41: 75.9 %)	*p* value^†^	Recurrence within Two years (*n* = 27: 35.5 %)	No recurrence for Two years (*n* = 49: 64.5 %)	*p* value^†^
Age						
Mean ± SD*	69.6 ± 5.4	65.8 ± 10.1	0.11	70.3 ± 7.3	67.3 ± 10.0	0.23
Sex						
Male	12 (92.3%)	29 (70.7%)		22 (81.5%)	39 (79.6%)	
Female	1 (7.7%)	12 (29.3%)	0.15	5 (18.5%)	10 (20.4%)	0.84
HBsAg						
Positive	2 (15.4%)	16 (39.0%)		0 (0%)	11 (22.4%)	
Negative	11 (84.6%)	25 (61.0%)	0.18	27 (100%)	38 (77.6%)	**0.0061**
HCVAb						
Positive	7 (53.8%)	17 (41.5%)		15 (55.6%)	25 (51.0%)	
Negative	6 (46.2%)	24 (58.5%)	0.43	12 (44.4%)	24 (49.0%)	0.7
Hepatitis virus infection						
Positive	9 (69.2%)	32 (78.0%)		15 (55.6%)	35 (71.4%)	
Negative	4 (30.8%)	9 (22.0%)	0.71	12 (44.6%)	14 (28.6%)	0.16
Platelet count (x10,000/μl)						
Mean ± SD*	14.4 ± 6.9	15.3 ± 5.1		16.5 ± 7.4	15.3 ± 5.6	
≤15	8 (61.5%)	19 (46.3%)		14 (51.8%)	21 (42.9%)	
>15	5 (38.5%)	22 (53.7%)	0.34	13 (48.2)	28 (57.1%)	0.45
Total bilirubin (mg/dl)						
Mean ± SD*	0.83 ± 0.26	0.78 ± 0.30		0.96 ± 0.35	0.81 ± 0.29	
≤0.7	6 (46.2%)	22 (53.7%)		8 (29.6%)	20 (40.8%)	
>0.7	7 (53.8%)	19 (46.3%)	0.64	19 (70.4%)	29 (59.2%)	0.33
Albumin (g/dl)						
Mean ± SD*	4.0 ± 0.32	4.2 ± 0.32		3.9 ± 0.37	4.1 ± 0.39	
<4.2	9 (69.2%)	17 (41.5%)		19 (70.4%)	30 (61.2%)	
≥4.2	4 (30.8%)	24 (58.5%)	0.081	8 (29.6%)	19 (38.8%)	0.43
Prothrombin time (%)						
Mean ± SD*	84.4 ± 22.1	94.6 ± 16.9		92.0 ± 15.6	94.1 ± 14.3	
≤93	9 (69.2%)	18 (43.9%)		14 (51.9%)	25 (51.0%)	
>93	4 (30.8%)	23 (56.1%)	0.11	13 (48.1%)	24 (49.0%)	0.94
ICGR15 (%)						
Mean ± SD*	21.2 ± 8.7	16.0 ± 7.0		17.6 ± 13.3	14.0 ± 6.7	
<15	5 (38.5%)	20 (48.8%)		11 (40.7%)	28 (57.1%)	
≥15	8 (61.5%)	21 (51.2%)	0.52	14 (51.9%)	21 (42.9%)	0.28
Unknown	–	–		2 (7.4%)	–	
Child‐Pugh classification						
A	13 (100 %)	41 (100%)		25 (92.6%)	47 (95.9%)	
B	0 (0 %)	0 (0%)	–	2 (7.4%)	2 (4.1%)	0.61
AFP (ng/ml)						
Median (range)	8.0 (2.3–250.3)	8.9 (1.9–6472)		12.7 (1.5–14425.4)	6.7 (1.5–25607.2)	
<10	8 (61.5%)	21 (51.2%)		12 (44.4%)	29 (59.2%)	
≥10	5 (38.5%)	20 (48.8%)	0.52	15 (55.6%)	20 (40.8%)	0.22
DCP (mAU/ml)						
Median (range)	79.0 (25–5099)	62.0 (2.3 ‐ 16153)		128 (13–357080)	28 (11–8249)	
<40	3 (23.1%)	17 (41.5%)		11 (40.7%)	27 (55.1%)	
≥40	10 (76.9%)	24 (58.5%)	0.33	16 (59.3%)	22 (44.9%)	0.23
Tumor size (mm)						
Mean ± SD*	29.2 ± 9.2	28.2 ± 9.3		31.6 ± 9.8	25.9 ± 12.1	
≤20	2 (15.4%)	10 (24.4%)		3 (11.1%)	17 (34.7%)	
20 < size ≤ 50	11 (84.6%)	31 (75.6%)	0.71	24 (88.9%)	32 (65.3%)	**0.026**
BCLC stage						
0	1 (7.7%)	8 (19.5%)		3 (11.1%)	16 (32.7%)	
A	12 (92.3%)	33 (80.5%)	0.43	24 (88.9%)	33 (67.3%)	**0.045**
Differentiation						
Well	2 (15.4%)	6 (14.6%)		3 (11.1%)	9 (18.4%)	
Moderately	9 (69.2%)	28 (68.3%)		22 (81.5%)	37 (75.5%)	
Poor	2 (15.4%)	7 (17.1%)	0.99	2 (7.4%)	3 (6.1%)	0.86
Background liver						
Non‐cirrhosis	7 (53.8%)	25 (61.0%)		19 (70.4%)	41 (83.7%)	
Cirrhosis	6 (46.2%)	16 (39.0%)	0.65	8 (29.6%)	8 (16.3%)	0.17
HBV : HCV : non‐B non‐C	1 : 3 : 2	6 : 9 : 1		0 : 7 : 1	2 : 3 : 3	
Hepatic resection						
Anatomical resection	8 (61.5%)	33 (80.5%)		14 (51.9%)	32 (65.3%)	
Non‐anatomical resection	5 (38.5%)	8 (19.5%)	0.26	13 (48.1%)	17 (34.7%)	0.25

Abbreviations: AFP, alpha‐fetoprotein; BCLC, the Barcelona Clinic Liver Cancer; DCP, des‐gamma‐carboxy prothrombin; ICGR15, indocyanine green retention rate at 15 min.

* SD: standard deviation.

^†^ *p* value is derived from chi‐square test, Fisher's exact test, Mann‐Whitney U test. Bold indicates a statistically significant.

We assessed the predictive potential of this three‐gene panel in the clinical training cohort (cohort‐1). Using a partial likelihood in CoxPH model, we obtained a risk scoring formula for the three‐gene panel as follows: (0.8777 x NSX24) + (0.2245 x HERC5) + (0.00483 x C1QTNF8). In this formula, we used ‐ΔCT values for determining the expression of each gene. The three‐gene panel demonstrated a significant predictive power in detecting EPR in early‐stage HCC patients (AUC = 0.82; Figure 2A, left panel). The waterfall plot with the risk scores and heatmap of gene expression for each patient is shown in Figure 2B (left panel). Cumulative recurrence rate analysis revealed that the high‐risk patients, categorized based upon our three‐gene panel, were associated with significantly higher recurrence rate in the clinical training cohort (*p *= 0.0004; Figure 2C, left panel). In univariate CoxPH regression analyses, our three‐gene panel emerged as the only significant predictor of early recurrence (HR 15.68, 95% CI 2.03–120.83, *p* = 0.0082; Figure 3A and Table S2) compared to all other clinical factors. In addition to our three‐gene panel, we included tumor size (high‐risk: tumor more than 2 cm)^7^ and operative procedure (high‐risk: non‐anatomical resection)^8^ in the multivariate CoxPH regression analyses because they are clinically important prognostic factors. In these analyses, our three‐gene panel emerged as the only independent significant predictor of EPR (HR 19.51, 95% CI 2.50–152.40, *p* = 0.0046; Figure 3A and Table S2).

**FIGURE 2:**
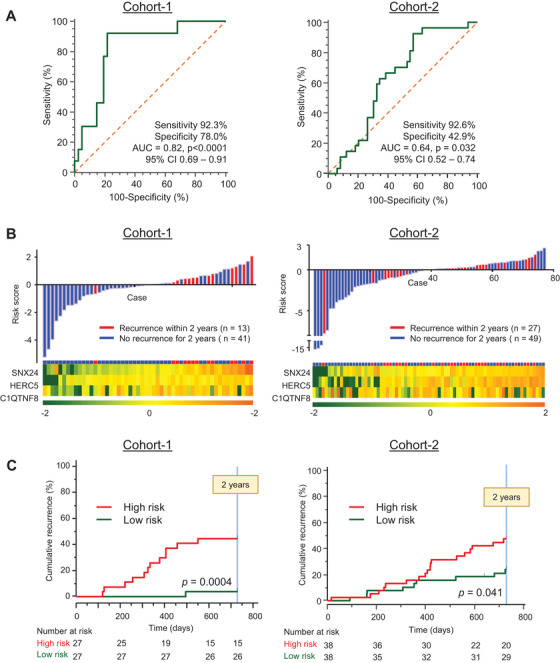
Diagnostic accuracy of three‐gene panel for predicting early phase recurrence in early‐stage HCC patients in clinical training cohort‐1 and clinical testing cohort‐2. (A) Receiver operating characteristic (ROC) curves for predicting early phase recurrence using three‐gene panel in both clinical cohorts. ROC curves are created by risk score based on a partial likelihood in Cox proportional hazard model. We used Youden's index for calculating sensitivity and specificity during ROC curve analysis. (B) Waterfall plot representing risk score of each patient generated from Cox proportional hazards model and a heatmap for three‐candidate genes in both clinical cohorts. We set the median of the risk scores to zero. Red and blue columns indicate patients with or without recurrence, respectively. (C) Cumulative recurrence rate curves for detecting 2‐year recurrence in using three‐gene panel. Patients in both clinical cohorts are stratified into high‐ and low‐risk using median expression values of the risk score panel score as cutoff thresholds. Red and green lines indicate high‐risk and low‐risk patients, respectively

**FIGURE 3:**
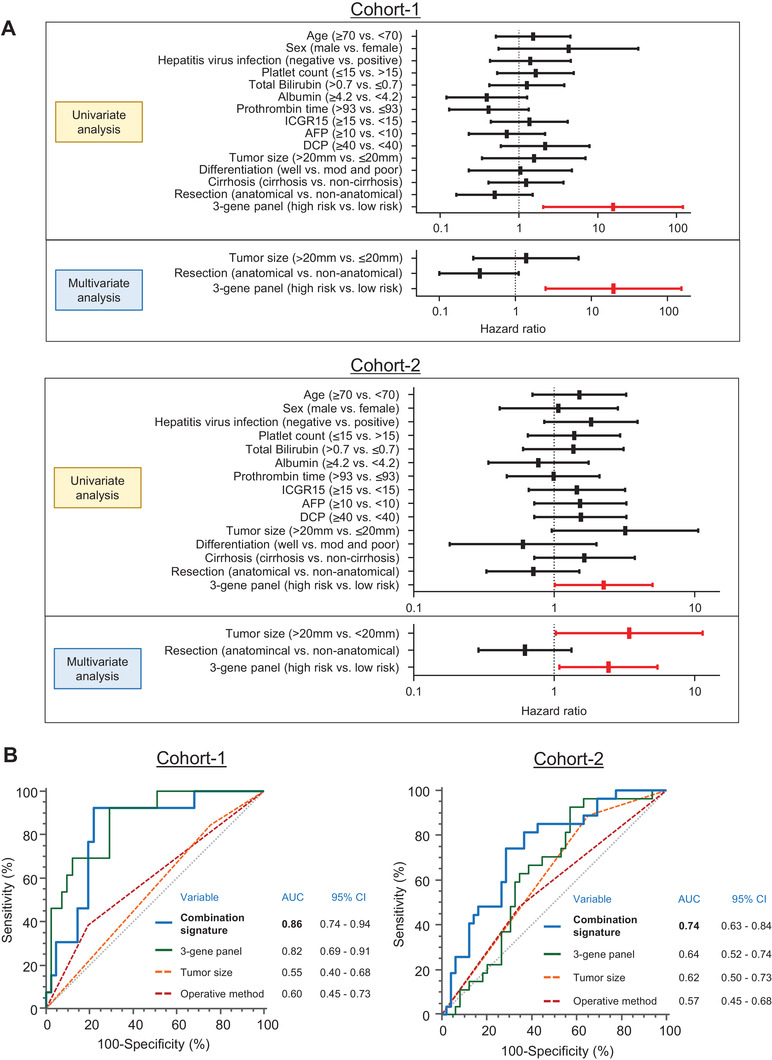
Predictive accuracy of combination signature with three‐gene panel and clinical factors in both clinical cohorts. (A) Forest plots representing univariate and multivariate analyses by Cox proportional hazards model in both clinical cohorts. (B) Comparison of receiver operating characteristic (ROC) curves of combination signature, three‐gene panel, tumor size, and operative method in clinical training cohort (cohort‐1) and testing cohort (cohort‐2), respectively

Subsequently, the predictive potential of this three‐gene panel was validated by applying the same risk‐score model and statistical correlates to the patients in the in the independent testing cohort (cohort‐2). As illustrated in Figure 2A (right panel), even in this cohort, our three‐gene panel was a significant predictor of EPR (AUC = 0.64). Similarly, Figure 2B (right panel), depicts the waterfall plot for the risk scores and heatmap of gene expression in cohort‐2 patients. The cumulative recurrence rates revealed that based upon our three‐gene panel, the high‐risk patients exhibited a significantly poorer prognosis compared to the low‐risk patients (*p *= 0.041; Figure 2C, right panel). Finally, the univariate CoxPH regression analysis using the three‐gene panel and various clinicopathological factors demonstrated that our gene‐panel was the only significant predictor of recurrence in patients within the testing cohort as well (HR 2.25, 95% CI 1.01 – 5.02, *p* = 0.047; Figure 3A and Table S2). In multivariate analyses using our three‐gene panel, tumor size, and operative method, the gene panel was the independent significant factor for predicting EPR in the testing cohort (HR 2.44, 95% CI 1.09 – 5.45, *p* = 0.030; Figure 3A and Table S2).

Next, we established a combination signature which included our three‐gene panel, tumor size, and operative method. This combination model was indeed superior versus individual factors and significantly improved the overall predictive accuracy in both cohort‐1 (AUC = 0.86; Figure 3B) and cohort‐2 patients (AUC = 0.74; Figure 3B). Taken together, our novel combination signature emerged as a potential signature that had significantly higher predictive value in predicting EPR in early‐stage HCC patients.

We would like to acknowledge a few potential limitations of our study. First, the tumor size (*p* = 0.0448) and total bilirubin (*p* = 0.0445) in the clinical cohort‐2 are not suitable for CoxPH analyses according to Schoenfeld residuals. Second, the performance of our three‐gene panel in the clinical cohort‐2 was not as robust, potentially due to the cohort size that was analyzed; hence, further clinical validation that includes larger prospective cohorts to assess the predictive accuracy of our three‐gene panel might be needed in future.

In summary, our genome‐wide, systematic biomarker discovery, and validation efforts resulted in the establishment of a novel three‐gene signature that could significantly predict EPR in patients with early‐stage HCCs; highlighting its potential clinical significance in the identification of high‐risk HCC patients undergoing surgical resection.

## CONFLICT OF INTEREST

The authors have no conflict of interest to disclose.

## FUNDING INFORMATION

This work was supported by CA72851, CA184792, and CA187956 grants from the National Institutes of Health/National Cancer Institute.

## AUTHOR CONTRIBUTIONS

Concept and design: Tatsuhiko Kakisaka and Ajay Goel. Acquisition, analysis, or interpretation of data: Tatsuhiko Kakisaka, Kensuke Yamamura, Takeo Toshima, and Ajay Goel. Drafting of the manuscript: Tatsuhiko Kakisaka and Ajay Goel. Statistical analysis: Tatsuhiko Kakisaka and Jasjit K. Banwait. Administrative, technical, or material support: Tatsuhiko Kakisaka, Moto Fukai, Tatsuhiko Kakisaka, Tatsuya Orimo, Tomoko Mitsuhashi, Kensuke Yamamura, Takeo Toshima, Hideo Baba, Akinobu Taketomi, and Ajay Goel. Supervision: Ajay Goel.

## Supporting information

SUPPORTING INFORMATION
