# Supplementary material for: Genomewide transcriptomic profiling identifies a gene signature for predicting recurrence in early‐stage hepatocellular carcinoma
Source: Clin Transl Med. 2021 Jun 6;11(6):e405. doi: 10.1002/ctm2.405 (PMC8181200; doi:10.1002/ctm2.405)
Supplement: Supplementary file 1 — SUPPORTING INFORMATION [file CTM2-11-e405-s001.docx]

**Genomewide transcriptomic profiling identifies a gene signature for predicting recurrence in early-stage hepatocellular carcinoma**

Tatsuhiko Kakisaka^1, 2^, Moto Fukai^3^, Jasjit K Banwait^1^, Toshiya Kamiyama^3^, Tatsuya Orimo^3^, Tomoko Mitsuhashi^4^, Kensuke Yamamura^5^, Takeo Toshima^6^, Hideo Baba^5^, Akinobu Taketomi^3^ and Ajay Goel^1, 2^

^1^Center for Gastrointestinal Research; Center from Translational Genomics and Oncology, Baylor Scott & White Research Institute and Charles A. Sammons Cancer Center, Baylor University Medical Center, Dallas, TX, USA.

^2^Department of Molecular Diagnostics and Experimental Therapeutics, Beckman Research Institute of City of Hope Comprehensive Cancer Center, Duarte, CA, USA

^3^ Department of Gastroenterological Surgery I, Graduate School of Medicine, Hokkaido University, Hokkaido, Japan.

^4^ Department of Surgical Pathology, Hokkaido University Hospital, Hokkaido, Japan.

^5^ Department of Gastroenterological Surgery, Graduate School of Medical Science, Kumamoto University, Kumamoto, Japan.

^6^ Department of Surgery and Science, Graduate School of Medical Sciences, Kyushu University, Fukuoka, Japan

**Supplementary data**

**
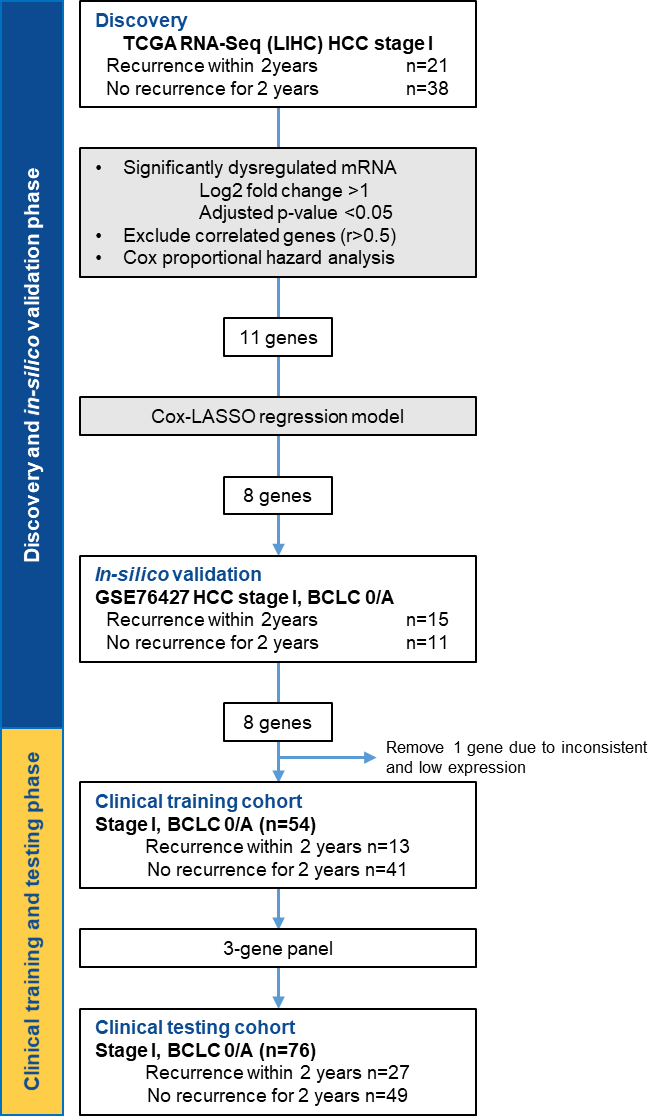
Figure S1:** Illustration of the Study design.

**
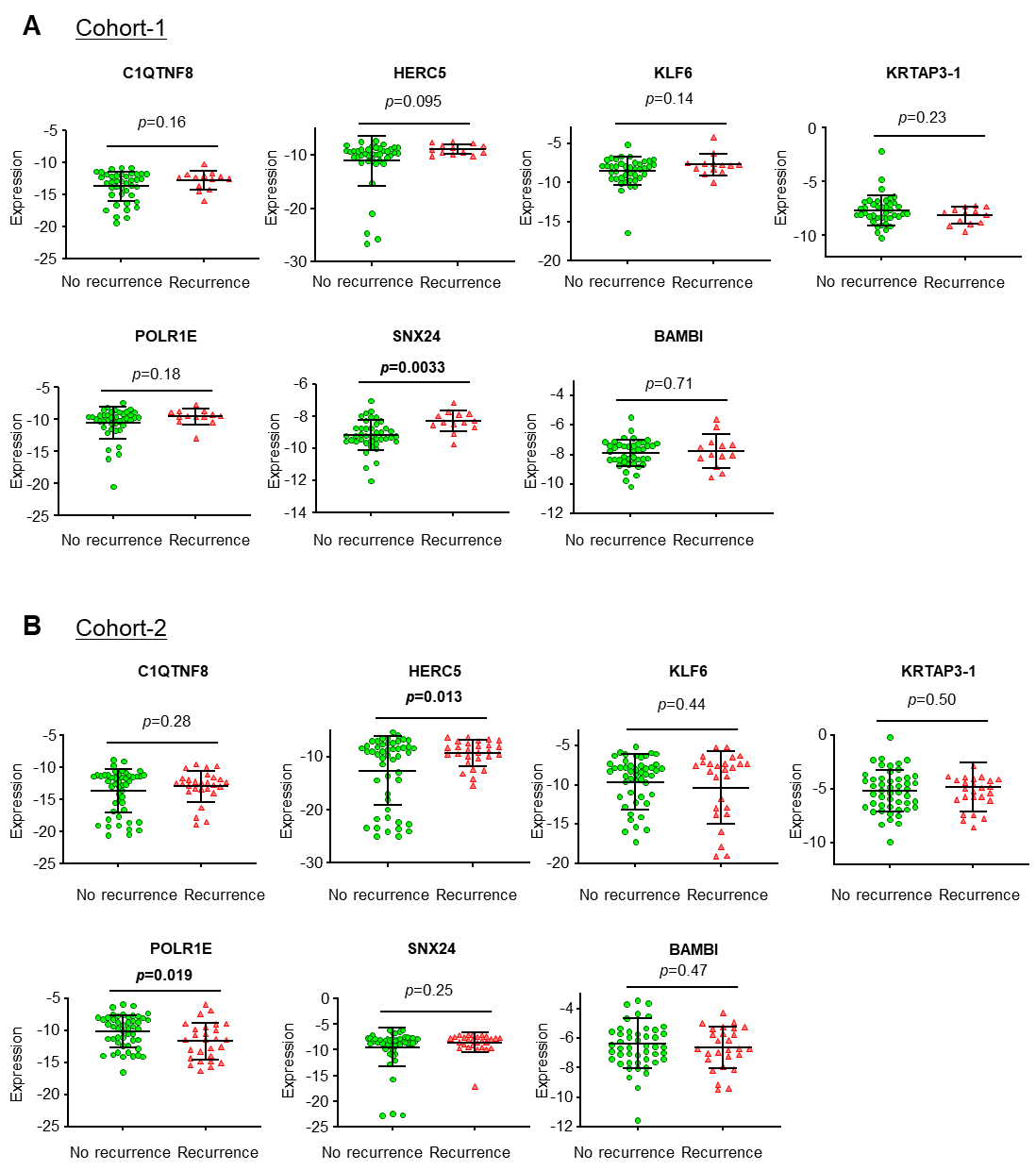
Figure S2:** Box plots indicating expression of seven genes in the clinical cohort-1 (A) and the clinical cohort-2 (B). Bold indicates a statistically significant by Student’s t-test. Y-axis is defined as -ΔCT.

**
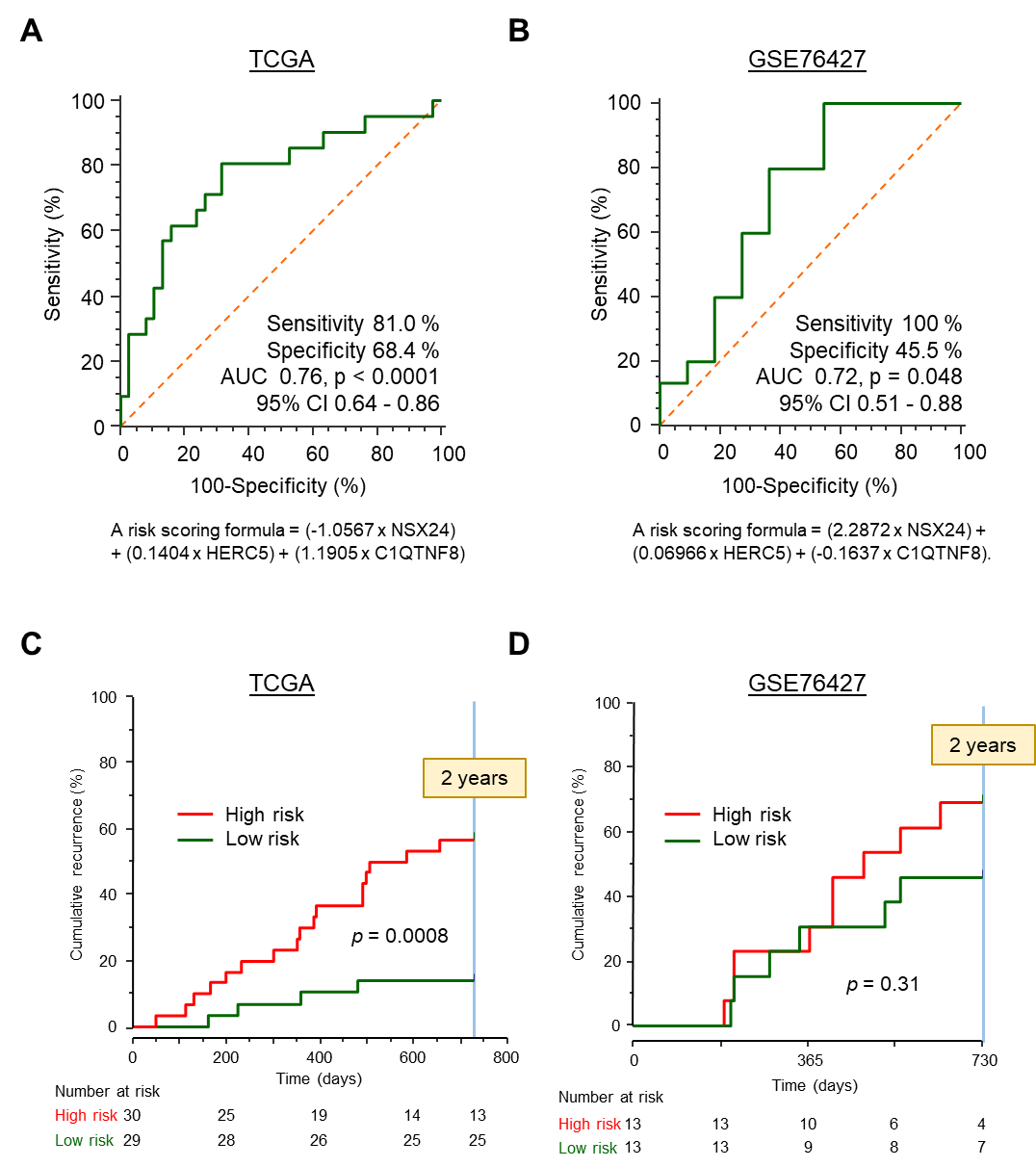
Figure S3:** Predictive value of 3-gene panel for identifying early phase recurrence in discovery and *in-silico* validation cohorts. (A) and (B) show receiver operating characteristic (ROC) curves of discovery dataset (TCGA) and *in-silico* validation dataset (GSE76427) for identifying early phase recurrence using 3-gene panel, respectively. ROC curves are created by risk score based on a partial likelihood in Cox proportional hazard model for both TCGA and GSE76427 datasets individually. We used Youden’s index for calculating sensitivity and specificity during ROC curve analysis. (C) and (D) show cumulative recurrence rate curves for detecting 2-year recurrence in TCGA cohort and GSE76427 cohort using 3-gene panel. Red and green lines indicate high-risk and low-risk patients, respectively. Patients in each cohort are stratified into high- and low risk using median expression values of individual 3-gene panel score as cutoff thresholds.

| **Table S1:** Primer pairs for 7 genes | | | | | | |  |
| --- | --- | --- | --- | --- | --- | --- | --- |
| Gene | Entrez ID | Forward primer | Tm | Reverse primer | Tm | Length of product | |
| KRTAP3-1 | 83896 | CTGTGCTCTCCGCTCCTG | 59.8 | GCAGCTTTTATCAAATGAGCAG | 56.6 | 70 | |
| SNX24 | 28966 | TCAGGGATCCATATGTCTTGC | 57.2 | GAGGACTCCTTCAATAACCACATT | 58.5 | 65 | |
| POLR1E | 64425 | TCAGGAACGTCACGTCAGAA | 59.0 | GGCAAAGACTTCAACGCTTC | 58.0 | 88 | |
| KLF6 | 1316 | CTCCCACTTGAAAGCACACC | 59.1 | ACTTCTTGCAAAACGCCACT | 58.9 | 91 | |
| HERC5 | 51191 | AGGGAATCAAAGCATTTTGC | 55.1 | CAGTCCCTTCATTCAGAGTAGGA | 59.0 | 89 | |
| C1QTNF8 | 390664 | TATGCCCGGGTGAGTGAC | 58.7 | CACCCTTCTCACCTTTGAGG | 57.8 | 106 | |
| BAMBI | 25805 | GGCTGCACGATGTTCTCTCT | 60.1 | AGGTTTCTGCTACCATCATGC | 58.4 | 82 | |

| **Table S2:** Univariate and multivariate analysis of the clinical cohort-1 and cohort-2 using Cox proportional hazard model | | | | | | | |
| --- | --- | --- | --- | --- | --- | --- | --- |
|  | Cohort-1 (n=54) | | |  | Cohort-2 (n=76) | | |
| Factor | HR | 95% CI | *p* |  | HR | 95% CI | *p* |
| **Univariate analysis** |  |  |  |  |  |  |  |
| Age |  |  |  |  |  |  |  |
| ≥70 vs. <70 | 1.51 | 0.51 - 4.49 | 0.46 |  | 1.51 | 0.70 - 3.26 | 0.29 |
| Sex |  |  |  |  |  |  |  |
| Male vs. female | 4.26 | 0.55 - 32.7 | 0.16 |  | 1.07 | 0.41 - 2.84 | 0.89 |
| Hepatitis virus infection |  |  |  |  |  |  |  |
| Negative vs. positive | 1.39 | 0.43 – 4.53 | 0.58 |  | 1.83 | 0.85 – 3.91 | 0.12 |
| Platelet count (x10,000/μl) |  |  |  |  |  |  |  |
| ≤15 vs. >15 | 1.63 | 0.53 - 4.97 | 0.39 |  | 1.39 | 0.65 - 2.95 | 0.4 |
| Total bilirubin (mg/dL) |  |  |  |  |  |  |  |
| >0.7 vs. ≤0.7 | 1.26 | 0.42 - 3.74 | 0.68 |  | 1.37 | 0.60 - 3.12 | 0.46 |
| Albumin (g/dL) |  |  |  |  |  |  |  |
| ≥4.2 vs. <4.2 | 0.39 | 0.12 - 1.27 | 0.29 |  | 0.55 | 0.25 - 1.21 | 0.14 |
| Prothrombin time (%) |  |  |  |  |  |  |  |
| 93> vs. ≤93 | 0.41 | 0.13 - 1.33 | 0.14 |  | 0.99 | 0.46 - 2.10 | 0.97 |
| ICGR15 (%) |  |  |  |  |  |  |  |
| ≥15 vs. <15 | 1.36 | 0.44 - 4.16 | 0.59 |  | 1.45 | 0.66 – 3.20 | 0.36 |
| AFP (ng/mL) |  |  |  |  |  |  |  |
| ≥10 vs. <10 | 0.70 | 0.23 - 2.14 | 0.53 |  | 1.53 | 0.72 - 3.27 | 0.27 |
| DCP (mAU/mL) |  |  |  |  |  |  |  |
| ≥40 vs. <40 | 2.15 | 0.59 - 7.83 | 0.24 |  | 1.55 | 0.72 - 3.34 | 0.26 |
| Tumor size (mm) |  |  |  |  |  |  |  |
| >20 vs. ≤20 | 1.55 | 0.34-6.99 | 0.57 |  | 3.20 | 0.96 – 10.62 | 0.058 |
| Differentiation |  |  |  |  |  |  |  |
| Well vs. moderate and poor | 1.04 | 0.23 - 4.71 | 0.96 |  | 0.60 | 0.18 - 2.00 | 0.41 |
| Cirrhosis |  |  |  |  |  |  |  |
| Cirrhosis vs. non-cirrhosis | 1.23 | 0.41 - 3.66 | 0.71 |  | 1.64 | 0.72 - 3.76 | 0.24 |
| Operative method |  |  |  |  |  |  |  |
| Anatomical vs. non-anatomical | 0.49 | 0.16 – 1.49 | 0.21 |  | 0.71 | 0.33 – 1.51 | 0.37 |
| 3-gene panel |  |  |  |  |  |  |  |
| High risk vs low risk | 15.68 | 2.03 - 120.83 | **0.0082** |  | 2.25 | 1.01 - 5.02 | **0.047** |
| **Multivariate analysis** |  |  |  |  |  |  |  |
| Tumor size (mm) |  |  |  |  |  |  |  |
| >20 vs. ≤20 | 1.39 | 0.28-6.79 | 0.69 |  | 3.43 | 1.03 – 11.41 | **0.045** |
| Operative method |  |  |  |  |  |  |  |
| Anatomical vs. non-anatomical | 0.34 | 0.10 – 1.11 | 0.074 |  | 0.62 | 0.29 – 1.33 | 0.22 |
| 3-gene panel |  |  |  |  |  |  |  |
| High risk vs low risk | 19.51 | 2.50 – 152.40 | **0.0046** |  | 2.44 | 1.09 – 5.45 | **0.030** |
| HR: hazard ratio, CI: confidence interval, Bold indicates a statistically significant. | | | | | | | |
| ICGR15: indocyanine green retention rate at 15 minutes, AFP: alpha-fetoprotein, DCP: des-gamma-carboxy prothrombin, | | | | | | | |
